# Causal associations of thyroid function with inflammatory bowel disease and the mediating role of cytokines

**DOI:** 10.3389/fendo.2024.1376139

**Published:** 2024-05-30

**Authors:** Shuyun Wu, Jiazhi Yi, Bin Wu

**Affiliations:** Department of Gastroenterology, The Third Affiliated Hospital of Sun Yat-sen University, Guangzhou, China

**Keywords:** thyroid function, hypothyroidism, inflammatory bowel disease, IP-10, Mendelian randomization

## Abstract

**Background:**

Previous observational epidemiological studies have suggested a potential association between thyroid function and inflammatory bowel disease (IBD). However, the findings remain inconclusive, and whether this association is causal remains uncertain. The objective of this study is to investigate the causal association between thyroid function and IBD.

**Methods:**

Genome-wide association studies (GWAS) involving seven indicators of thyroid function, IBD, and 41 cytokines were analyzed. Bidirectional two-sample Mendelian randomization (MR) and multivariable MR were conducted to examine the causal relationship between thyroid function and IBD and to explore the potential mechanisms underlying the associations.

**Results:**

Genetically determined hypothyroidism significantly reduced the risk of CD (odds ratio [OR] = 0.761, 95% CI: 0.655–0.882, *p* < 0.001). Genetically determined reference-range TSH was found to have a suggestive causal effect on IBD (OR = 0.931, 95% CI: 0.888–0.976, *p* = 0.003), (Crohn disease) CD (OR = 0.915, 95% CI: 0.857–0.977, *p* = 0.008), and ulcerative colitis (UC) (OR =0.910, 95% CI: 0.830–0.997, *p* = 0.043). In reverse MR analysis, both IBD and CD appeared to have a suggestive causal effect on the fT3/fT4 ratio (OR = 1.002, *p* = 0.013 and OR = 1.001, *p* = 0.015, respectively). Among 41 cytokines, hypothyroidism had a significant impact on interferon-inducible protein-10 (IP-10) (OR = 1.465, 95% CI: 1.094–1.962, *p* = 0.010). The results of multivariable MR showed that IP-10 may mediate the causal effects of hypothyroidism with CD.

**Conclusion:**

Our results suggest that an elevated TSH level reduces the risk of CD, with IP-10 potentially mediating this association. This highlights the pituitary-thyroid axis could serve as a potential therapeutic strategy for CD.

## 1 Background

Inflammatory bowel disease (IBD) is a chronic and nonspecific inflammatory disorder of the intestinal tract, encompassing Crohn’s disease (CD) and ulcerative colitis (UC) (1). Despite significant advances in understanding IBD, the exact etiology of the disease remains largely uncertain. A substantial body of literature suggests that the pathogenesis of IBD is linked to intestinal microbiota, a specific genetic background, environmental factors, and abnormal immune responses (2). Although IBD primarily affects the gastrointestinal tract, patients may experience extraintestinal manifestations in various organs, such as the blood, liver, pancreas, prostate, cervix uteri, central nervous system, and skin (3). The severity of certain extraintestinal symptoms may vary depending on the underlying activity of IBD (4).

The regulation of thyroid function is a complex process that involves not only the thyroid gland but also the pituitary gland and the hypothalamus. Thyrotropin (TSH), secreted by the pituitary, stimulates the thyroid to release thyroxine (T4). In thyroidal and peripheral tissues, free T4 is converted to free triiodothyronine (fT3) hormone to fulfill physiological functions (5, 6). Autoimmunity is the predominant etiology of thyroid dysfunction, encompassing both hyperthyroidism (7) and hypothyroidism (8). Autoimmune thyroid disorders, including Grave’s disease, Hashimoto thyroiditis, and postpartum thyroiditis, are characterized by circulating thyroid-specific autoreactive antibodies (9).

Recent studies suggest that thyroid function extends beyond thyroid diseases to include links to the gastrointestinal system (10, 11). The gut microbiota contributes to the synthesis and hydrolysis of thyroid hormone conjugates. Microbial metabolites could potentially contribute to autoimmune thyroid diseases by modulating the immune response (12, 13). Several studies have explored the relationship between thyroid diseases and IBD, yielding variable and even conflicting results. A cross-sectional study conducted in England, enrolling 300 UC patients, identified a significant increase in the prevalence of thyrotoxicosis (14). Additionally, several studies indicate that IBD patients may have an increased susceptibility to thyroid gland carcinogenesis (15, 16). Modifications in thyroid gland size and function, both with and without clinically detectable hyperthyroidism or hypothyroidism, have been reported in IBD patients (17). Nevertheless, the exact role of thyroid hormones in the pathophysiology of IBD remains unclear. It remains uncertain whether the observed connection between thyroid function and IBD is causal.

Mendelian randomization (MR) is a method frequently employed to explore causal links between risk factors and outcomes using genetic instruments (18). Our study conducted a bidirectional two-sample MR analysis to investigate the causal relationship between thyroid function and IBD, including ulcerative colitis (UC) and Crohn’s disease (CD) subtypes. Cytokines play a critical role in the pathophysiological processes of both IBD and autoimmune thyroid disorders. Furthermore, we conducted a multivariable MR analysis to assess whether cytokines mediate the causal relationship between thyroid function and IBD.

## 2 Materials and methods

### 2.1 Study design

**Figure 1:**
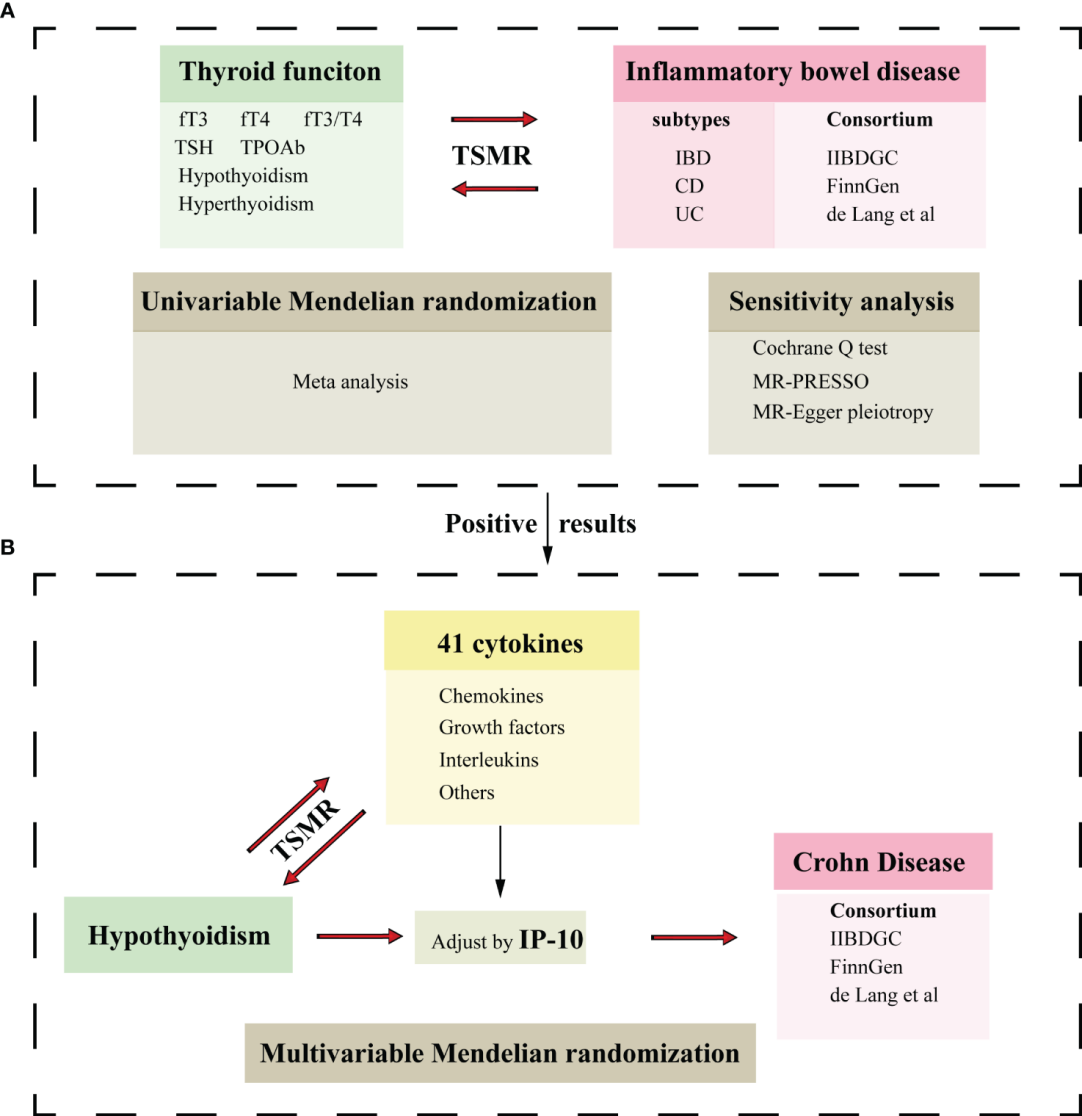
Flowchart of the Mendelian randomization study. **(A)** Two-sample Mendelian randomization investigates the effect of thyroid function on IBD and its subtypes.**(B)** Two-sample Mendelian randomization investigates the effect of hypothyroidism on cytokines and multivariable Mendelian randomization analysis evaluates the roles of mediating the association between thyroid function and CD. IBD, inflammatory bowel disease; UC, ulcerative colitis; CD, Crohn’s disease; fT3, free triiodothyronine; fT4, free thyroxine; TSH, thyroid stimulating hormone; TPOAb, thyroid peroxidase antibody; IP-10, interferon-inducible protein-10; TSMR, Two-sample Mendelian randomization.

We conducted a bidirectional two-sample MR study to investigate the causal relationship between thyroid function and IBD. The flowchart is displayed in **Figure 1**.

GWAS summary data for thyroid functions included reference-range free triiodothyronine (fT3), free thyroxine (fT4), the ratio of fT3 to fT4 (fT3/fT4), thyrotropin (TSH), thyroid peroxidase antibody (TPOAb) positivity, decreased TSH status (indicative of hyperthyroidism), and increased TSH status (indicative of hypothyroidism). Genome-wide association study (GWAS) summary data for IBD included both UC and CD subtypes. Subsequently, we performed a two-sample MR analysis to explore the causal effects of both hyperthyroidism and hypothyroidism on cytokines. A multivariable MR analysis determined the effect of hypothyroidism on CD after adjusting for interferon-inducible protein-10 (IP-10). The selection of instrumental variables (IVs) must adhere to three fundamental principles: (1) relevance assumption—the genetic variation is highly associated with exposure; (2) independence assumption—the genetic variation is not significantly linked to potential confounding factors; and (3) exclusion restriction assumption—the genetic variation exclusively affects the outcome through the exposure.

### 2.2 Data sources

In this study, we utilized summary-level data from an updated meta-analysis of GWAS conducted by the ThyroidOmics Consortium (19). GWAS data for TPOAb positivity were acquired from (20). GWAS data for IBD were obtained from the International IBD Genetics Consortium (IIBDGC) (21), the FinnGen database, and a large-scale GWAS study (22). The GWAS data for cytokines was derived from the meta-analysis summary statistics for 41 inflammatory cytokines (23). Detailed information about the datasets used in this study is presented in **Supplementary Table 1**. All summary statistics employed were from GWAS analyses, and no sample overlap was observed. All GWAS summary data are publicly available; thus, no additional ethical approval or informed consent was required.

### 2.3 Genetic variants selection criteria

Single nucleotide polymorphisms (SNPs) meeting genome-wide significance (*p* < 5 × 10^−8^) and having minor allele frequency (MAF) >1% were included. Due to the scarcity of SNPs with p-values less than 5×10^−8^, we extended the threshold to 1×10^−5^ for TPOAb positivity and 5×10^−6^ for cytokines to select appropriate instrumental variables. The identified SNPs were then clumped with a strict cutoff of clumping R^2^ = 0.001 within a window of 10,000 kb. The proportions of trait variance explained by the identified SNPs were calculated using the following formulas: R^2^ = (2β^2^ × MAF × (1 - MAF))/(2β^2^ × MAF × (1 - MAF) + 2 N × MAF × (1-MAF) × SE^2^), where MAF is the minor allele frequency, β is the effect estimate of the SNP in the exposure GWAS, SE is the standard error, and N is the sample size. Additionally, we assessed instrument strength using the F statistic, defined as F = (R^2^ × (N - 2))/(1 - R^2^), to evaluate the significant association of the genetic instruments with the exposure (24, 25).

### 2.4 Statistical analysis

The inverse variance weighted (IVW) method was employed as the primary approach in our MR analysis, providing accurate estimates in the absence of heterogeneity and directional pleiotropy between the exposure and outcome (26). The heterogeneity of the IVW model was assessed using Cochran’s Q test. If significant heterogeneity was indicated by Cochran’s Q test (*p* < 0.05), we transitioned from the fixed inverse variance-weighted model to the random-effects model. Additionally, the MR Egger method was utilized to estimate the causal effect, with the capability to identify and adjust for any directional pleiotropy. The MR Pleiotropy RESidual Sum and Outlier (MR-PRESSO) method was applied to assess horizontal pleiotropy (27). If horizontal pleiotropy was detected, it was corrected by removing the outlier and assessing whether substantial variations in the causal effects existed before and after outlier removal. Furthermore, the MR-Egger regression intercept term was employed to evaluate the potential presence of horizontal pleiotropy, where a deviation from zero (*p* < 0.05) suggests directional pleiotropy (28). Finally, a meta-analysis was conducted to assess the combined causality between thyroid function and IBD from MR results across various databases. The choice of effect model depended on the degree of heterogeneity observed. For minimal heterogeneity (I^2^ ≤ 50%), the fixed-effects model was applied. For substantial heterogeneity (I^2^ > 50%), the random-effects model was utilized. The findings of the meta-analysis were considered the definitive evidence of causality.

All MR analyses adhered to the guidelines outlined in the STROBE-MR Statement (29). For the MR analysis examining the relationship between thyroid function and IBD, we applied a Bonferroni-corrected significance threshold, calculated as 0.0024 (0.05 divided by 21, accounting for 7 exposures and 3 outcomes). P-values between 0.0024 and 0.05 were considered indicative of potential causal associations between the exposures and outcomes. For the MR analysis between hyperthyroidism/hypothyroidism and cytokines, a p-value of less than 0.05 was considered statistically significant. All statistical analyses were conducted using the “TwoSample MR” package (version 0.5.6) in R software (version 4.2.2), and data visualization was also performed in R.

## 3 Results

### 3.1 Genetic instruments

After screening based on the corresponding p-values and linkage disequilibrium (LD) clumping, we calculated the variance explained by the genetic instruments. We quantified the instrument strength by calculating the F-statistics for each SNP, noting that a value of 10 or higher indicates adequate strength and the absence of bias from weak instruments. The number and specific characteristics of SNPs selected for each thyroid function phenotype and IBD are detailed in **Supplementary Table 2** and **Supplementary Table 3**.

### 3.2 The causal effect of thyroid function on IBD, CD and UC.

**Figure 2:**
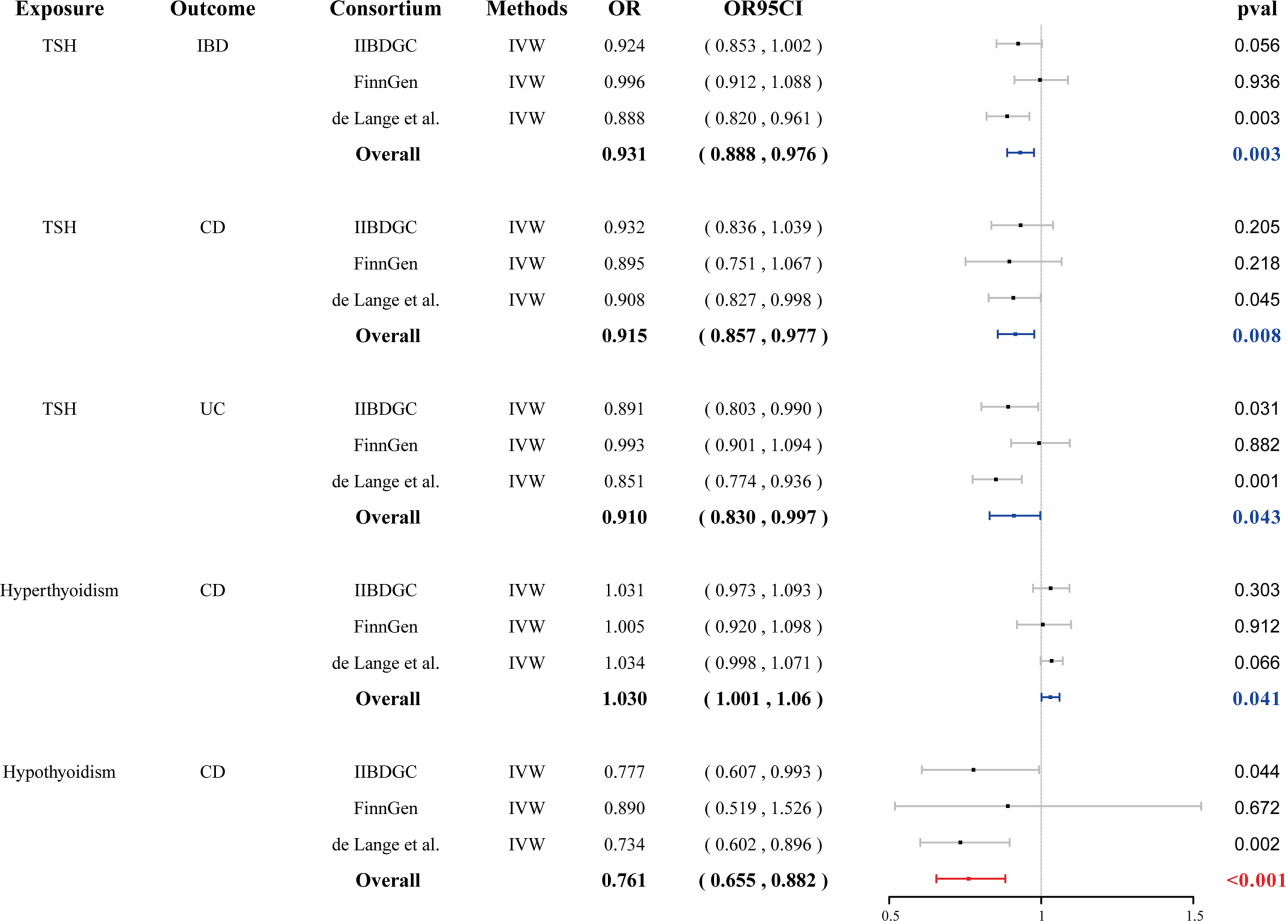
Forest plots of the association between seven thyroid function indexes on IBD and final causality. fT3, free triiodothyronine; fT4, free thyroxine; TSH, thyroid stimulating hormone; TPOAb, thyroid peroxidase antibody; IBD, inflammatory bowel disease; UC, ulcerative colitis, CD, Crohn’s disease; IVW, inverse-variance weighted; OR, odds ratio.

We found that genetically predicted hypothyroidism was associated with a reduced risk of CD (OR = 0.761, 95% CI: 0.655–0.882, *p* < 0.001). Genetically determined hyperthyroidism has a suggestive causal effect on CD (OR = 1.030, 95% CI: 1.001–1.061, *p* = 0.041) (**Figure 2**). No evidence of pleiotropy or heterogeneity was detected in the MR-PRESSO global test, MR-Egger intercept test, and Cochran’s Q test. Genetically determined TSH within the reference range has a suggestive causal effect on IBD (OR = 0.931, 95% CI: 0.888–0.976, *p* = 0.003), CD (OR = 0.915, 95% CI: 0.857–0.977, *p* = 0.008) and UC (OR =0.910, 95% CI: 0.830–0.997, *p* = 0.043). Little heterogeneity and no pleiotropy were observed. No causal association was observed between fT3, fT4, the ratio of fT4/fT3, and TPOAb positivity with IBD, CD, and UC (**Supplementary Figures S1–S3**, **Supplementary Table 4** and **Supplementary Table 5**).

### 3.3 The causal effect of IBD, CD and UC on thyroid function.

**Figure 3:**
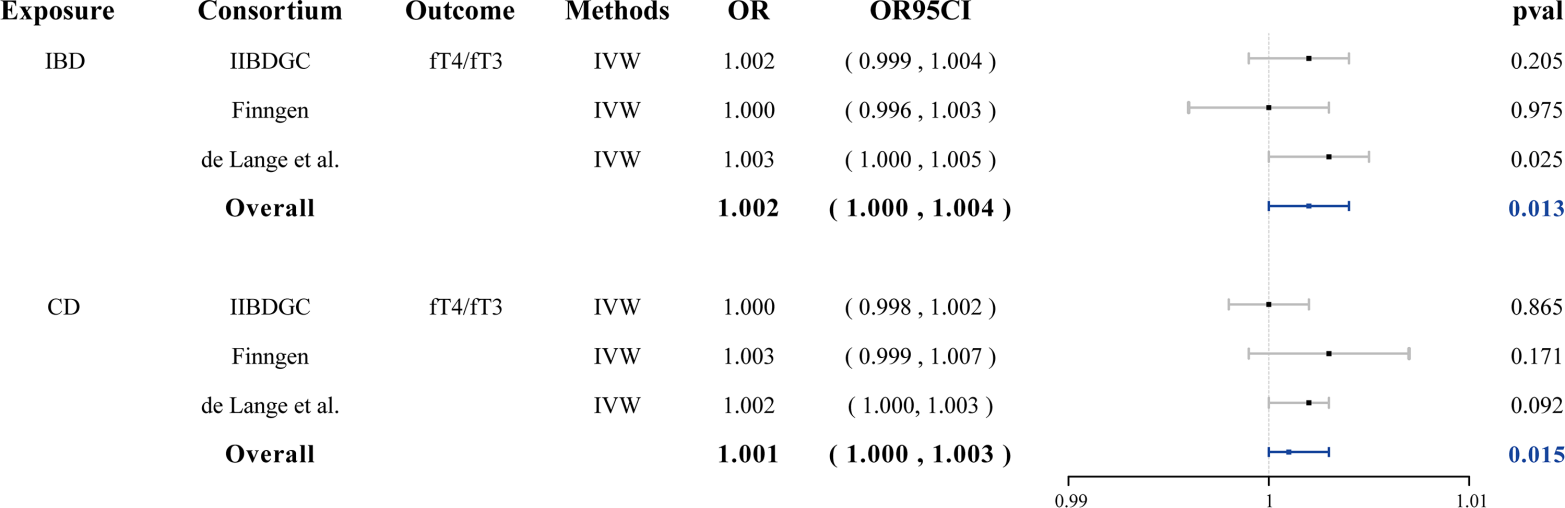
Forest plots of the association between IBD/CD on fT3/fT4 ratio and final causality. fT3, free triiodothyronine; fT4, free thyroxine; IBD, inflammatory bowel disease; CD, Crohn’s disease; IVW, inverse-variance weighted; OR, odds ratio.

In reverse MR analysis, both IBD and CD demonstrated a suggestive causal effect on the fT3/fT4 ratio (OR = 1.002, 95% CI: 1.000–1.004, *p* = 0.013 and OR = 1.001, 95% CI: 1.000–1.003, *p* = 0.015, respectively) (**Figure 3**). No evidence of pleiotropy or heterogeneity was detected. Genetically predicted UC was not causally associated with the fT3/fT4 ratio. Genetically predicted IBD, CD, and UC were not causally associated with fT3, fT4, TPOAb positivity, TSH, hyperthyroidism, and hypothyroidism. (**Supplementary Figures S4–S6**, **Supplementary Table 6** and **Supplementary Table 7**).

### 3.4 The causal effect of hypothyroidism and hyperthyroidism on cytokines

**Figure 4:**
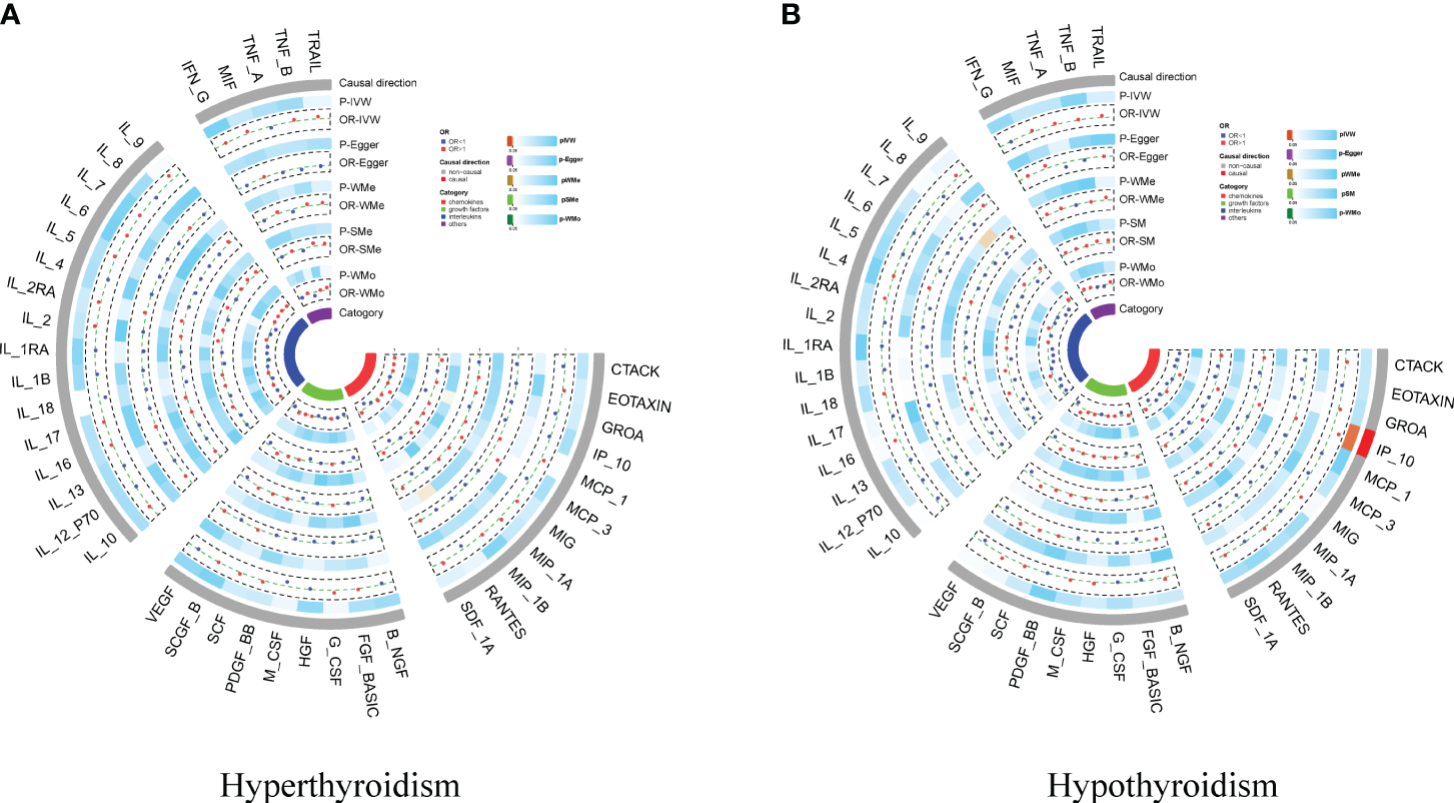
Circo heatmap of the effect of hypothyroidism/hyperthyroidism on 41 cytokines. **(A)** the effect of hyperthyroidism on 41 cytokines; **(B)** the effect of hypothyroidism on 41 cytokines.

We conducted a two-sample MR to investigate the causal effects of hyperthyroidism and hypothyroidism on cytokines. We found no significant causal association between hypothyroidism and cytokines overall; however, interferon gamma-induced protein 10 (IP-10) showed an association (OR = 1.465, 95% CI:1.094–1.962, *p* = 0.010. No causal relationship was observed between hyperthyroidism and cytokines (**Figures 4A, B**; **Supplementary Table 8** and **Supplementary Table 9**). Reverse Mendelian randomization did not reveal an association between IP-10 and hypothyroidism. We identified a significant causal association between interleukin-2 (IL-2) and stem cell growth factor beta (SCGF-β) with hyperthyroidism (OR = 1.134, 95% CI: 1.037–1.241, *p* = 0.006; OR = 1.106, 95% CI: 1.019–1.202, *p* = 0.017, respectively). The presence of interleukin-13 (IL-13) and macrophage migration inhibitory factor (MIF) are causally associated with hyperthyroidism (OR = 1.046, 95% CI: 1.008–1.086, *p* = 0.019; OR = 1.052, 95% CI: 1.007–1.099, p = 0.022, respectively) (**Supplementary Figure S7**, **Supplementary Table 10** and **Supplementary Table 11**).

### 3.5 Multivariable MR

**Figure 5:**
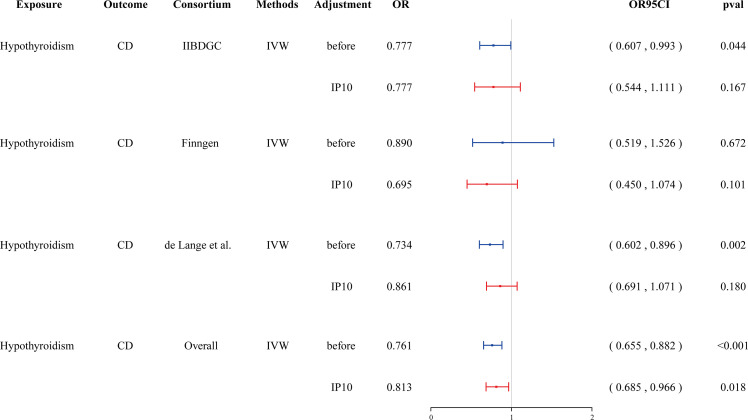
Forest plot showing the effect of hypothyroidism on CD after adjustment in Multivariate MR. CD, Crohn’s disease; IVW, inverse-variance weighted; IP-10, interferon-inducible protein-10.

Cytokines are involved in both thyroid function disorders and IBD. We hypothesized that cytokines might mediate the causal relationship between hypothyroidism and CD. Given that hypothyroidism is only causally associated with IP-10, we incorporated IP-10 to conduct a multivariable MR. We found that the association between hypothyroidism and CD disappeared after adjusting for IP-10 in the GWAS databases of the IIBDGC and de Lang et al. The meta-analysis further demonstrated that the association between hypothyroidism and CD was not significant (OR = 0.813, 95% CI: 0.685–0.966, *p* = 0.018, failing to meet the Bonferroni-corrected significance threshold of 0.0024) (**Figure 5**, **Supplementary Table 12**). This suggests that IP-10 may mediate the causal effect of hypothyroidism on CD.

## 4 Discussion

In this MR study, we demonstrated that hypothyroidism exerts an inverse causal effect on CD but not on IBD or UC. Additionally, we found that TSH within the reference range was suggestive of causality associated with IBD and its subtypes, while hyperthyroidism was suggestive of correlation with CD. In the reverse-directional MR, both IBD and CD demonstrated suggestive associations with the ratio of fT4/fT3.

The relationship between thyroid disorders (TDs) and IBD has been explored in numerous studies, yielding variable and occasionally conflicting results. Snook et al. (30) reported a positive association between extraintestinal autoimmune diseases and UC regardless of thyroid function status, but this association was not observed in CD. Similarly, a recent population-based study of 8,072 IBD patients identified an increased risk of TDs in UC patients aged 40 to 59 years, while no such risk was identified in CD patients (31). Casella et al. (32) observed a significantly lower prevalence of thyroid dysfunction among UC patients compared to the general Italian population. In contrast, another study found that individuals with UC experienced a markedly higher occurrence of thyroid dysfunction, two to four times more prevalent than in the general population (33). In a case-control study, Ricart et al. (34) observed a generally lower frequency of autoimmune diseases, particularly autoimmune thyroid disease, among IBD patients. A retrospective cross-sectional study, limited to individuals with CD, demonstrated only a statistically marginal reduction in the prevalence of hypothyroidism, whereas no significant change was observed in hyperthyroidism (35). A retrospective, single-center, case-control study indicated that patients with IBD have a reduced likelihood of developing thyroid disorders (36). Routine hormonal assessments and thyroid gland imaging may not be necessary in the absence of clinical signs or symptoms. According to their research findings, the development of thyroid disorders may exhibit either a positive or negative correlation with the onset of IBD or its subtypes. Even studies highlighting a positive association of IBD with increased extraintestinal autoimmunity, when compared to non-IBD individuals, noted that this phenomenon was limited to rheumatoid arthritis and dermatological disorders, excluding common autoimmune diseases such as autoimmune thyroiditis (37). Interestingly, even among IBD patients with a first-degree family history of disease, the prevalence of autoimmune diseases was not increased. Extensive research in Israel involving 12,967 individuals with IBD showed a notable increase in the prevalence of several autoimmune diseases, with the remarkable exception of TDs (38). Our investigation revealed that both IBD and CD exhibited a suggestive causal association with fT3/fT4, indicating a potential causal link between IBD and thyroid dysfunction to some extent.

Intestinal fibrosis, which results in stricture formation and obstruction, represents a significant complication of CD. Although therapeutic management of CD has improved with novel agents, an effective approach to address CD-related stricture continues to be elusive. Recent research has indicated that thyroid function is associated with fibrosis-related diseases, such as lung fibrosis (39, 40) and liver fibrosis (41, 42). Thyroid hormone has demonstrated efficacy in inhibiting lung fibrosis by enhancing epithelial mitochondrial function (43). Triiodothyronine (T3) potentially enhances the resolution of pulmonary fibrosis and inhibits fibroblast activation and extracellular matrix production. Additionally, T3 regulates the interaction between macrophages and fibroblasts (44). In liver fibrosis, thyroid hormone regulates the activation of hepatic stellate cells through thyroid hormone receptor α and modulation of transforming growth factor downstream signaling (45). Fibrotic stenosis of the intestinal lumen resulting from CD shares some similarities with pulmonary fibrosis and hepatic fibrosis, as all are linked to inflammation. Consequently, thyroid hormone could represent a potential therapeutic target for CD-related stricture.

A substantial body of research has documented the higher prevalence of thyroid dysfunction in IBD patients compared to the general population. However, investigations into the impact of thyroid function on IBD from alternative perspectives remain limited. Thyroid hormones not only affect gastrointestinal motor function (46) but also play a crucial role in maintaining intestinal epithelial homeostasis. Recent studies indicate that thyroid hormones and their receptors perform diverse functions in intestinal stem cells and their niches (47). Furthermore, the well-documented bidirectional crosstalk between thyroid hormones and the immune response underscores their interplay (48, 49). Studies have demonstrated that T3 elevates the number of IL-17-expressing T lymphocytes by activating dendritic cells *in vitro* (50). Circulating TH levels positively correlate with immunological reactivity in healthy individuals, supporting the physiological maintenance of lymphocyte subpopulations (51). Hyperthyroidism is associated with enhanced humoral and immune cell responses (49). Conversely, hypothyroidism is linked to contrary effects (52). Jaeger et al. (53) identified a significant correlation between TSH concentrations and various populations of effector and regulatory T cells through analysis of the immunological phenotype from the Human Functional Genomics Project. Serum IL-27 levels are elevated in subjects with hypothyroidism and inversely correlate with the incidence of nonalcoholic fatty liver disease (54). Hypothyroidism is linked to immunosuppression, which is attributed to increased frequency and activity of Gal-1-expressing Tregs, with significant implications for immunopathology, metabolic disorders, and cancer (55). Apart from the pituitary, T and B lymphocytes can synthesize and release TSH, potentially affecting both healthy and abnormal thyroid cells expressing the TSH receptor (56, 57). This novel and unexpected non-pituitary source of TSH could also be decisive in affecting the immune response during infections and chronic inflammation.

Interferon-inducible protein-10 (IP-10), also referred to as CXCL10, is a chemokine essential for the activation of integrins and migration of cells, including activated T cells, monocytes, eosinophils, NK cells, epithelial and endothelial cells (58). The IP-10/CXCR3 axis is instrumental in monocyte activation and elicits a Th1 response to facilitate effector cell recruitment in inflamed intestinal tissues (59, 60). In experimental murine models, treatment with anti-IP-10 antibodies has been shown to protect against epithelial ulceration and reduce inflammation by impairing Th1 induction and recruitment (61). BMS-936557 (previously known as MDX-1100) is a fully humanized monoclonal antibody that targets IP-10. A phase II study has demonstrated the safety and potential efficacy of BMS-936557 in UC patients (62). Our MR analysis has determined an inverse causal effect of hypothyroidism on CD but not on IBD and UC, potentially IP-10-dependent. Xian et al. (63) conducted an MR analysis examining the relationship between Grave’s disease (GD) and IBD. Their findings suggest that IBD and CD may increase the risk of GD, while UC may provide a protective effect against the development of GD. Conversely, GD may slightly increase the risk of CD. However, this research underscores an ethnic disparity in terms of exposure and outcome, which could potentially explain the divergent findings observed by our team.

This study faces several constraints and limitations. A major limitation of this investigation is the lack of comprehensive GWAS in non-European ancestries, which necessitates reliance on studies conducted in European populations to estimate the causal effects. Future studies should focus on the assessment of these causal pathways in diverse ethnic groups. Another limitation stems from the absence of an association between abnormal fT3 and fT4 levels and IBD, a consequence of the constraints inherent in GWAS datasets. Regrettably, our study did not conduct subgroup analysis by gender for hypo- and hyperthyroidism, conditions that are more prevalent in females than in males. The physiological and biochemical impacts of TSH on CD require further validation via cellular and animal experiments.

## 5 Conclusions

This study demonstrated that hypothyroidism had an impact on CD, potentially mediated by IP-10. This discovery suggests approaches for more effective prevention and intervention of CD. However, it is important to acknowledge that these findings are based on genetic prediction and necessitate further validation through subsequent research.

## Data availability statement

The original contributions presented in the study are included in the article/**Supplementary Material**, further inquiries can be directed to the corresponding author/s.

## Author contributions

SW: Methodology, Formal analysis, Data curation, Writing – original draft. JY: Writing – original draft, Methodology, Formal analysis, Data curation. BW: Writing – review & editing, Supervision, Investigation, Funding acquisition.

## References

[1] Lamb CA Kennedy NA Raine T Hendy PA Smith PJ Limdi JK. British Society of Gastroenterology consensus guidelines on the management of inflammatory bowel disease in adults. Gut. (2019) 68:s1–s106. doi: 10.1136/gutjnl-2019-318484 31562236 PMC6872448

[2] Guan Q. A comprehensive review and update on the pathogenesis of inflammatory bowel disease. J Immunol Res. (2019) 2019:7247238. doi: 10.1155/2019/7247238 31886308 PMC6914932

[3] Harbord M Annese V Vavricka SR Allez M Barreiro-de Acosta M Boberg KM. The first european evidence-based consensus on extra-intestinal manifestations in inflammatory bowel disease. J Crohns Colitis. (2016) 10:239–54. doi: 10.1093/ecco-jcc/jjv213 PMC4957476 26614685

[4] Rogler G Singh A Kavanaugh A Rubin DT. Extraintestinal manifestations of inflammatory bowel disease: current concepts, treatment, and implications for disease management. Gastroenterology. (2021) 161:1118–32. doi: 10.1053/j.gastro.2021.07.042 PMC8564770 34358489

[5] Chen J Bi H Pettersson ME Sato DX Fuentes-Pardo AP Mo C. Functional differences between TSHR alleles associate with variation in spawning season in Atlantic herring. Commun Biol. (2021) 4:795. doi: 10.1038/s42003-021-02307-7 34172814 PMC8233318

[6] Kopp P. The TSH receptor and its role in thyroid disease. Cell Mol Life Sci. (2001) 58:1301–22. doi: 10.1007/PL00000941 PMC11337400 11577986

[7] Lee SY Pearce EN. Hyperthyroidism: A review. Jama. (2023) 330:1472–83. doi: 10.1001/jama.2023.19052 PMC10873132 37847271

[8] Chaker L Razvi S Bensenor IM Azizi F Pearce EN Peeters RP. Hypothyroidism. Nat Rev Dis Primers. (2022) 8:30. doi: 10.1038/s41572-022-00357-7 35589725

[9] Ferrari SM Fallahi P Elia G Ragusa F Ruffilli I Paparo SR. Thyroid autoimmune disorders and cancer. Semin Cancer Biol. (2020) 64:135–46. doi: 10.1016/j.semcancer.2019.05.019 31158464

[10] Fenneman AC Bruinstroop E Nieuwdorp M van der Spek AH Boelen A. A comprehensive review of thyroid hormone metabolism in the gut and its clinical implications. Thyroid. (2023) 33:32–44. doi: 10.1089/thy.2022.0491 36322786

[11] Kyriacou A McLaughlin J Syed AA. Thyroid disorders and gastrointestinal and liver dysfunction: A state of the art review. Eur J Intern Med. (2015) 26:563–71. doi: 10.1016/j.ejim.2015.07.017 26260744

[12] Ejtahed HS Angoorani P Soroush AR Siadat SD Shirzad N Hasani-Ranjbar S. Our little friends with big roles: alterations of the gut microbiota in thyroid disorders. Endocr Metab Immune Disord Drug Targets. (2020) 20:344–50. doi: 10.2174/1871530319666190930110605 31566142

[13] Knezevic J Starchl C Tmava Berisha A Amrein K. Thyroid-gut-axis: how does the microbiota influence thyroid function? Nutrients. (2020) 12:1769. doi: 10.3390/nu12061769 32545596 PMC7353203

[14] Jarnerot G Azad Khan AK Truelove SC. The thyroid in ulverative colitis and Crohn's disease. II. Thyroid enlargement and hyperthyroidism in ulcerative colitis. Acta Med Scand. (1975) 197:83–7. doi: 10.1111/j.0954-6820.1975.tb04882.x 1124663

[15] Levi Z Fraser E Krongrad R Hazazi R benjaminov O meyerovitch J. Factors associated with radiation exposure in patients with inflammatory bowel disease. Aliment Pharmacol Ther. (2009) 30:1128–36. doi: 10.1111/j.1365-2036.2009.04140.x 19899197

[16] Wadhwa V Lopez R Shen B. Crohn's disease is associated with the risk for thyroid cancer. Inflammation Bowel Dis. (2016) 22:2902–6. doi: 10.1097/MIB.0000000000000963 27846192

[17] Bianchi GP Marchesini G Gueli C Zoli M. Thyroid involvement in patients with active inflammatory bowel diseases. Ital J Gastroenterol. (1995) 27:291–5. 8562993

[18] Bowden J Holmes MV. Meta-analysis and Mendelian randomization: A review. Res Synth Methods. (2019) 10:486–96. doi: 10.1002/jrsm.1346 PMC6973275 30861319

[19] Teumer A Chaker L Groeneweg S Li Y Di Munno C Barbieri C. Genome-wide analyses identify a role for SLC17A4 and AADAT in thyroid hormone regulation. Nat Commun. (2018) 9:4455. doi: 10.1038/s41467-018-06356-1 30367059 PMC6203810

[20] Medici M Porcu E Pistis G Teumer A Brown SJ Jensen RA. Identification of novel genetic Loci associated with thyroid peroxidase antibodies and clinical thyroid disease. PloS Genet. (2014) 10:e1004123. doi: 10.1371/journal.pgen.1004123 24586183 PMC3937134

[21] Liu JZ van Sommeren S Huang H Ng SC Alberts R Takahashi A. Association analyses identify 38 susceptibility loci for inflammatory bowel disease and highlight shared genetic risk across populations. Nat Genet. (2015) 47:979–86. doi: 10.1038/ng.3359 PMC4881818 26192919

[22] de Lange KM Moutsianas L Lee JC Lamb CA Luo Y Kennedy NA. Genome-wide association study implicates immune activation of multiple integrin genes in inflammatory bowel disease. Nat Genet. (2017) 49:256–61. doi: 10.1038/ng.3760 PMC5289481 28067908

[23] Ahola-Olli AV Wurtz P Havulinna AS Aalto K Pitkanen N Lehtimaki T. Genome-wide association study identifies 27 loci influencing concentrations of circulating cytokines and growth factors. Am J Hum Genet. (2017) 100:40–50. doi: 10.1016/j.ajhg.2016.11.007 27989323 PMC5223028

[24] Palmer TM Lawlor DA Harbord RM Sheehan NA Tobias JH Timpson NJ. Using multiple genetic variants as instrumental variables for modifiable risk factors. Stat Methods Med Res. (2012) 21:223–42. doi: 10.1177/0962280210394459 PMC3917707 21216802

[25] Morales Berstein F McCartney DL Lu AT Tsilidis KK Bouras E Haycock P. Assessing the causal role of epigenetic clocks in the development of multiple cancers: a Mendelian randomization study. Elife. (2022) 11:e75374. doi: 10.7554/eLife.75374 35346416 PMC9049976

[26] Thompson JR Minelli C Abrams KR Tobin MD Riley RD. Meta-analysis of genetic studies using Mendelian randomization–a multivariate approach. Stat Med. (2005) 24:2241–54. doi: 10.1002/(ISSN)1097-0258 15887296

[27] Verbanck M Chen CY Neale B Do R. Detection of widespread horizontal pleiotropy in causal relationships inferred from Mendelian randomization between complex traits and diseases. Nat Genet. (2018) 50:693–8. doi: 10.1038/s41588-018-0099-7 PMC6083837 29686387

[28] Bowden J Davey Smith G Burgess S. Mendelian randomization with invalid instruments: effect estimation and bias detection through Egger regression. Int J Epidemiol. (2015) 44:512–25. doi: 10.1093/ije/dyv080 PMC4469799 26050253

[29] Burgess S Davey Smith G Davies NM Dudbridge F Gill D Glymour MM. Guidelines for performing Mendelian randomization investigations: update for summer 2023. Wellcome Open Res. (2019) 4:186. doi: 10.12688/wellcomeopenres 32760811 PMC7384151

[30] Snook JA de Silva HJ Jewell DP. The association of autoimmune disorders with inflammatory bowel disease. Q J Med. (1989) 72:835–40. 2616728

[31] Bernstein CN Wajda A Blanchard JF. The clustering of other chronic inflammatory diseases in inflammatory bowel disease: a population-based study. Gastroenterology. (2005) 129:827–36. doi: 10.1053/j.gastro.2005.06.021 16143122

[32] Casella G De Marco E Antonelli E Daperno M Baldini V Signorini S. The prevalence of hyper- and hypothyroidism in patients with ulcerative colitis. J Crohns Colitis. (2008) 2:327–30. doi: 10.1016/j.crohns.2008.09.001 21172232

[33] Bonapace ES Srinivasan R. Simultaneous occurrence of inflammatory bowel disease and thyroid disease. Am J Gastroenterol. (2001) 96:1925–6. doi: 10.1111/j.1572-0241.2001.03896.x 11419852

[34] Ricart E Panaccione R Loftus EV Jr. Tremaine WJ Harmsen WS Zinsmeister AR. Autoimmune disorders and extraintestinal manifestations in first-degree familial and sporadic inflammatory bowel disease: a case-control study. Inflammation Bowel Dis. (2004) 10:207–14. doi: 10.1097/00054725-200405000-00005 15290913

[35] Pooran N Singh P Bank S. Crohn's disease and risk of fracture: does thyroid disease play a role? World J Gastroenterol. (2003) 9:615–8. doi: 10.3748/wjg.v9.i3.615 PMC4621595 12632531

[36] Dore MP Fanciulli G Manca A Cocco V Nieddu A Murgia M. Clinically relevant thyroid disorders and inflammatory bowel disease are inversely related: a retrospective case-control study. Scand J Gastroenterol. (2021) 56:171–6. doi: 10.1080/00365521.2020.1861323 33327797

[37] Wilson JC Furlano RI Jick SS Meier CR. Inflammatory bowel disease and the risk of autoimmune diseases. J Crohns Colitis. (2016) 10:186–93. doi: 10.1093/ecco-jcc/jjv193 26507860

[38] Bar Yehuda S Axlerod R Toker O Zigman N Goren I Mourad V. The association of inflammatory bowel diseases with autoimmune disorders: A report from the epi-IIRN. J Crohns Colitis. (2019) 13:324–9. doi: 10.1093/ecco-jcc/jjy166 30304371

[39] Zhang J Zhang L Chen Y Fang X Li B Mo C. The role of cGAS-STING signaling in pulmonary fibrosis and its therapeutic potential. Front Immunol. (2023) 14:1273248. doi: 10.3389/fimmu.2023.1273248 37965345 PMC10642193

[40] Ma J Li G Wang H Mo C. Comprehensive review of potential drugs with anti-pulmonary fibrosis properties. BioMed Pharmacother. (2024) 173:116282. doi: 10.1016/j.biopha.2024.116282 38401514

[41] Puengel T Tacke F. Cell type-specific actions of thyroid hormones in nonalcoholic steatohepatitis and liver fibrosis. Liver Int. (2024) 44:275–8. doi: 10.1111/liv.15783 38289588

[42] Yan M Li H Xu S Wu J Li J Xiao C. Targeting endothelial necroptosis disrupts profibrotic endothelial-hepatic stellate cells crosstalk to alleviate liver fibrosis in nonalcoholic steatohepatitis. Int J Mol Sci. (2023) 24:11313. doi: 10.3390/ijms241411313 37511074 PMC10379228

[43] Yu G Tzouvelekis A Wang R Herazo-Maya JD Ibarra GH Srivastava A. Thyroid hormone inhibits lung fibrosis in mice by improving epithelial mitochondrial function. Nat Med. (2018) 24:39–49. doi: 10.1038/nm.4447 29200204 PMC5760280

[44] Wang L Li Z Wan R Pan X Li B Zhao H. Single-cell RNA sequencing provides new insights into therapeutic roles of thyroid hormone in idiopathic pulmonary fibrosis. Am J Respir Cell Mol Biol. (2023) 69:456–69. doi: 10.1165/rcmb.2023-0080OC PMC10557923 37402274

[45] Manka P Coombes JD Sydor S Swiderska-Syn MK Best J Gauthier K. Thyroid hormone receptor alpha modulates fibrogenesis in hepatic stellate cells. Liver Int. (2024) 44:125–38. doi: 10.1111/liv.15759 37872645

[46] Nakazawa N Sohda M Ogata K Baatar S Ubukata Y Kuriyama K. Thyroid hormone activated upper gastrointestinal motility without mediating gastrointestinal hormones in conscious dogs. Sci Rep. (2021) 11:9975. doi: 10.1038/s41598-021-89378-y 33976260 PMC8113274

[47] Giolito MV Plateroti M. Thyroid hormone signaling in the intestinal stem cells and their niche. Cell Mol Life Sci. (2022) 79:476. doi: 10.1007/s00018-022-04503-y 35947210 PMC11072102

[48] Klecha AJ Genaro AM Lysionek AE Caro RA Coluccia AG Cremaschi GA. Experimental evidence pointing to the bidirectional interaction between the immune system and the thyroid axis. Int J Immunopharmacol. (2000) 22:491–500. doi: 10.1016/S0192-0561(00)00012-6 10785546

[49] De Vito P Incerpi S Pedersen JZ Luly P Davis FB Davis PJ. Thyroid hormones as modulators of immune activities at the cellular level. Thyroid. (2011) 21:879–90. doi: 10.1089/thy.2010.0429 21745103

[50] Alamino VA Montesinos MDM Soler MF Giusiano L Gigena N Fozzatti L. Dendritic cells exposed to triiodothyronine deliver pro-inflammatory signals and amplify IL-17-driven immune responses. Cell Physiol Biochem. (2019) 52:354–67. doi: 10.33594/000000000 30816679

[51] Hodkinson CF Simpson EE Beattie JH O'Connor JM Campbell DJ Strain JJ. Preliminary evidence of immune function modulation by thyroid hormones in healthy men and women aged 55-70 years. J Endocrinol. (2009) 202:55–63. doi: 10.1677/JOE-08-0488 19398496

[52] Klecha AJ Barreiro Arcos ML Frick L Genaro AM Cremaschi G. Immune-endocrine interactions in autoimmune thyroid diseases. Neuroimmunomodulation. (2008) 15:68–75. doi: 10.1159/000135626 18667802

[53] Jaeger M Sloot YJE Horst RT Chu X Koenen H Koeken V. Thyrotrophin and thyroxine support immune homeostasis in humans. Immunology. (2021) 163:155–68. doi: 10.1111/imm.13306 PMC8114202 33454989

[54] Wen Y Zhang H Yang N Gao X Chen Z Liu J. Serum IL-27 levels increase in subjects with hypothyroidism and are negatively correlated with the occurrence of nonalcoholic fatty liver disease. Front Endocrinol (Lausanne). (2023) 14:1173826. doi: 10.3389/fendo.2023.1173826 37600722 PMC10433777

[55] Valli E Dalotto-Moreno T Sterle HA Mendez-Huergo SP Paulazo MA Garcia SI. Hypothyroidism-associated immunosuppression involves induction of galectin-1-producing regulatory T cells. FASEB J. (2023) 37:e22865. doi: 10.1096/fj.202200884R 36934391

[56] Smith EM Phan M Kruger TE Coppenhaver DH Blalock JE. Human lymphocyte production of immunoreactive thyrotropin. Proc Natl Acad Sci U.S.A. (1983) 80:6010–3. doi: 10.1073/pnas.80.19.6010 PMC534349 6351072

[57] Harbour DV Kruger TE Coppenhaver D Smith EM Meyer WJ. Differential expression and regulation of thyrotropin (TSH) in T cell lines. Mol Cell Endocrinol. (1989) 64:229–41. doi: 10.1016/0303-7207(89)90150-0 2507375

[58] Rotondi M Chiovato L Romagnani S Serio M Romagnani P. Role of chemokines in endocrine autoimmune diseases. Endocr Rev. (2007) 28:492–520. doi: 10.1210/er.2006-0044 17475924

[59] Zhao Q Kim T Pang J Sun W Yang X Wang J. A novel function of CXCL10 in mediating monocyte production of proinflammatory cytokines. J Leukoc Biol. (2017) 102:1271–80. doi: 10.1189/jlb.5A0717-302 28899907

[60] Trivedi PJ Adams DH. Chemokines and chemokine receptors as therapeutic targets in inflammatory bowel disease; pitfalls and promise. J Crohns Colitis. (2018) 12:S641–52. doi: 10.1093/ecco-jcc/jjx145 PMC6104621 30137309

[61] Hyun JG Lee G Brown JB Grimm GR Tang Y Mittal N. Anti-interferon-inducible chemokine, CXCL10, reduces colitis by impairing T helper-1 induction and recruitment in mice. Inflammation Bowel Dis. (2005) 11:799–805. doi: 10.1097/01.MIB.0000178263.34099.89 16116313

[62] Mayer L Sandborn WJ Stepanov Y Geboes K Hardi R Yellin M. Anti-IP-10 antibody (BMS-936557) for ulcerative colitis: a phase II randomised study. Gut. (2014) 63:442–50. doi: 10.1136/gutjnl-2012-303424 PMC3933070 23461895

[63] Xian W Wu D Liu B Hong S Huo Z Xiao H. Graves disease and inflammatory bowel disease: A bidirectional mendelian randomization. J Clin Endocrinol Metab. (2023) 108:1075–83. doi: 10.1210/clinem/dgac683 PMC10099169 36459455

