# Correction: Causal associations of thyroid function with inflammatory bowel disease and the mediating role of cytokines

**DOI:** 10.3389/fendo.2025.1647808

**Published:** 2025-07-01

**Authors:** Shuyun Wu, Jiazhi Yi, Bin Wu

**Affiliations:** Department of Gastroenterology, The Third Affiliated Hospital of Sun Yat-sen University, Guangzhou, China

**Keywords:** thyroid function, hypothyroidism, inflammatory bowel disease, IP-10, Mendelian randomization

In the published article, there was an error in the article title. Instead of “Casual associations of thyroid function with inflammatory bowel disease and the mediating role of cytokines”, it should be “Causal associations of thyroid function with inflammatory bowel disease and the mediating role of cytokines”.

The original article has been updated.

