# Supplementary material for: Causal associations of thyroid function with inflammatory bowel disease and the mediating role of cytokines
Source: Front Endocrinol (Lausanne). 2024 May 30;15:1376139. doi: 10.3389/fendo.2024.1376139 (PMC11169666; doi:10.3389/fendo.2024.1376139)
Supplement: Supplementary file 2 [file DataSheet_1.docx]

**supplementary information**

**Supplementary Table1** Description of GWAS summary statitics used for this study.

**Supplementary Table 2** Number of genome-wide significant index SNPs for exposure in the study.

**Supplementary Table 3** Genome-wide significant index SNPs for this study

**Supplementary Table 4** Causal effects of thyroid function on IBD, CD and UC.

**Supplementary Table 5** The summary of causal effects, heterogeneity and pleiotropy of thyroid function on IBD, CD and UC.

**Supplementary Table 6** Causal effects of IBD, CD and UC on thyroid function

**Supplementary Table 7** The summary of causal effects, heterogeneity and pleiotropy of IBD, CD and UC on thyroid function

**Supplementary Table 8** The summary of causal effects, heterogeneity and pleiotropy of hyperthyroidism on cytokines

**Supplementary Table 9** The summary of causal effects, heterogeneity and pleiotropy of hypothyroidism on cytokines.

**Supplementary Table 10** The summary of causal effects, heterogeneity and pleiotropy of cytokines on hyperthyroidism.

**Supplementary Table 11** The summary of causal effects, heterogeneity and pleiotropy of cytokines on hypothyroidism.

**Supplementary Table 12** Multivariable MR of hypothyroidism on CD after adjusting for TNF-α, IL-10, IL-17, IP-10.

**Supplementary Figure 1** Forest plots of the association between thyroid function on IBD and final causality.

**Supplementary Figure 2** Forest plots of the association between thyroid function on CD and final causality.

**Supplementary Figure 3** Forest plots of the association between thyroid function on UC and final causality.

**Supplementary Figure 4** Forest plots of the association between IBD on thyroid function and final causality.

**Supplementary Figure 5** Forest plots of the association between IBD on thyroid function and final causality.

**Supplementary Figure 6** Forest plots of the association between IBD on thyroid function and final causality.

**Supplementary Figure 7** Circo heatmap of the effect of 41 cytokines on hypothyroidism/hyperthyroidism.
