# Supplementary figures and images for: Causal associations of thyroid function with inflammatory bowel disease and the mediating role of cytokines
Source: Front Endocrinol (Lausanne). 2024 May 30;15:1376139. doi: 10.3389/fendo.2024.1376139 (PMC11169666; doi:10.3389/fendo.2024.1376139)

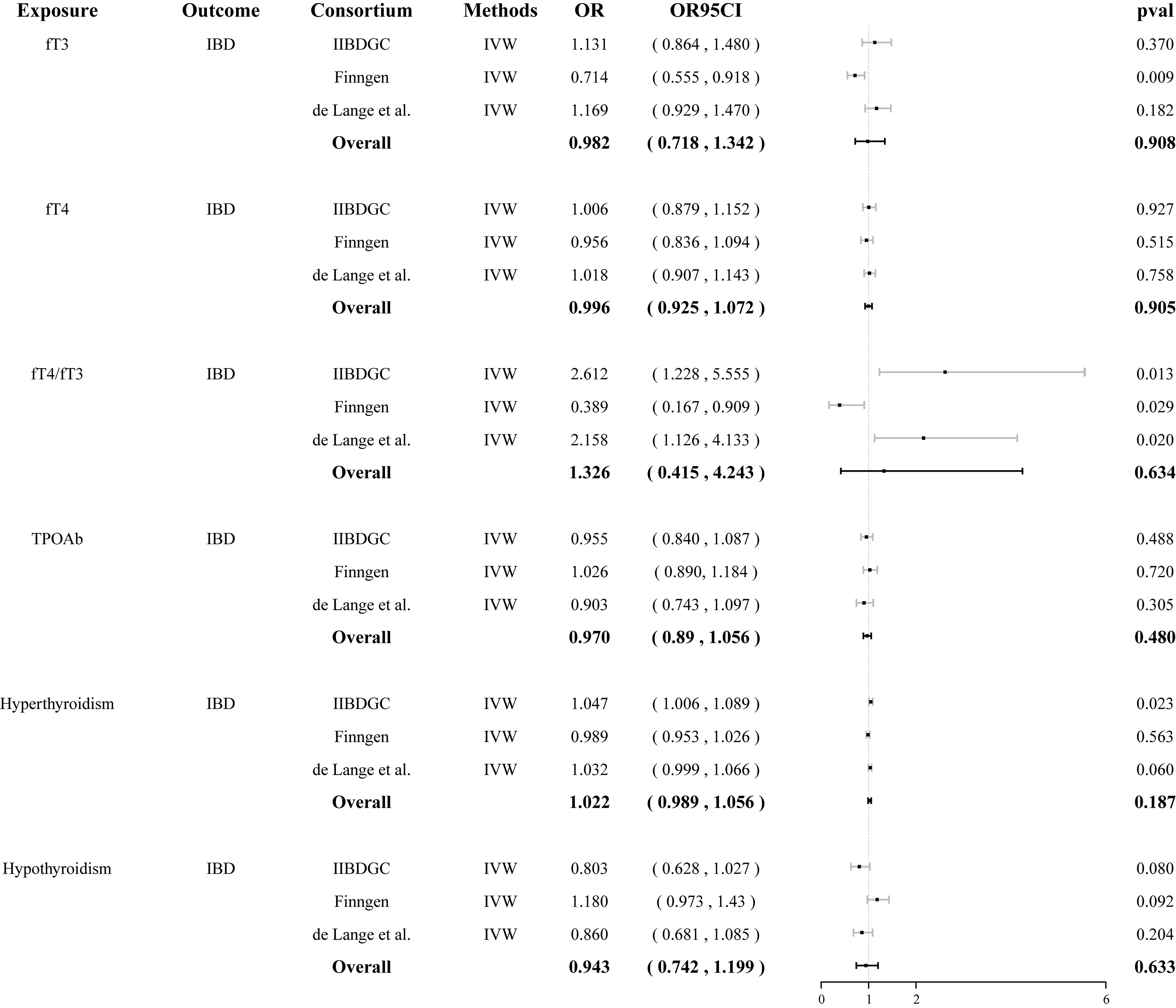

Supplement: Supplementary file 3 [file Image_1.tif]

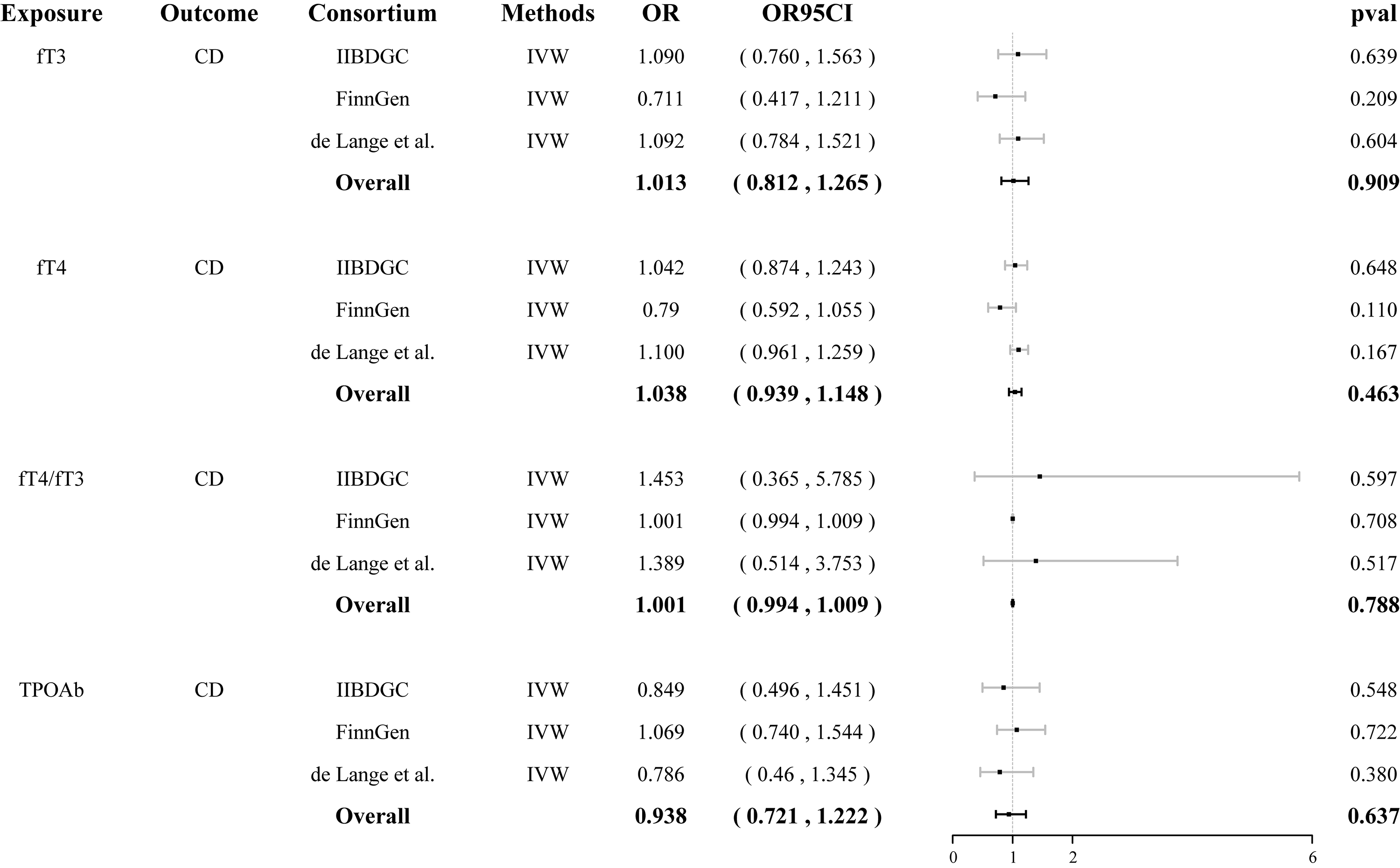

Supplement: Supplementary file 4 [file Image_2.tif]

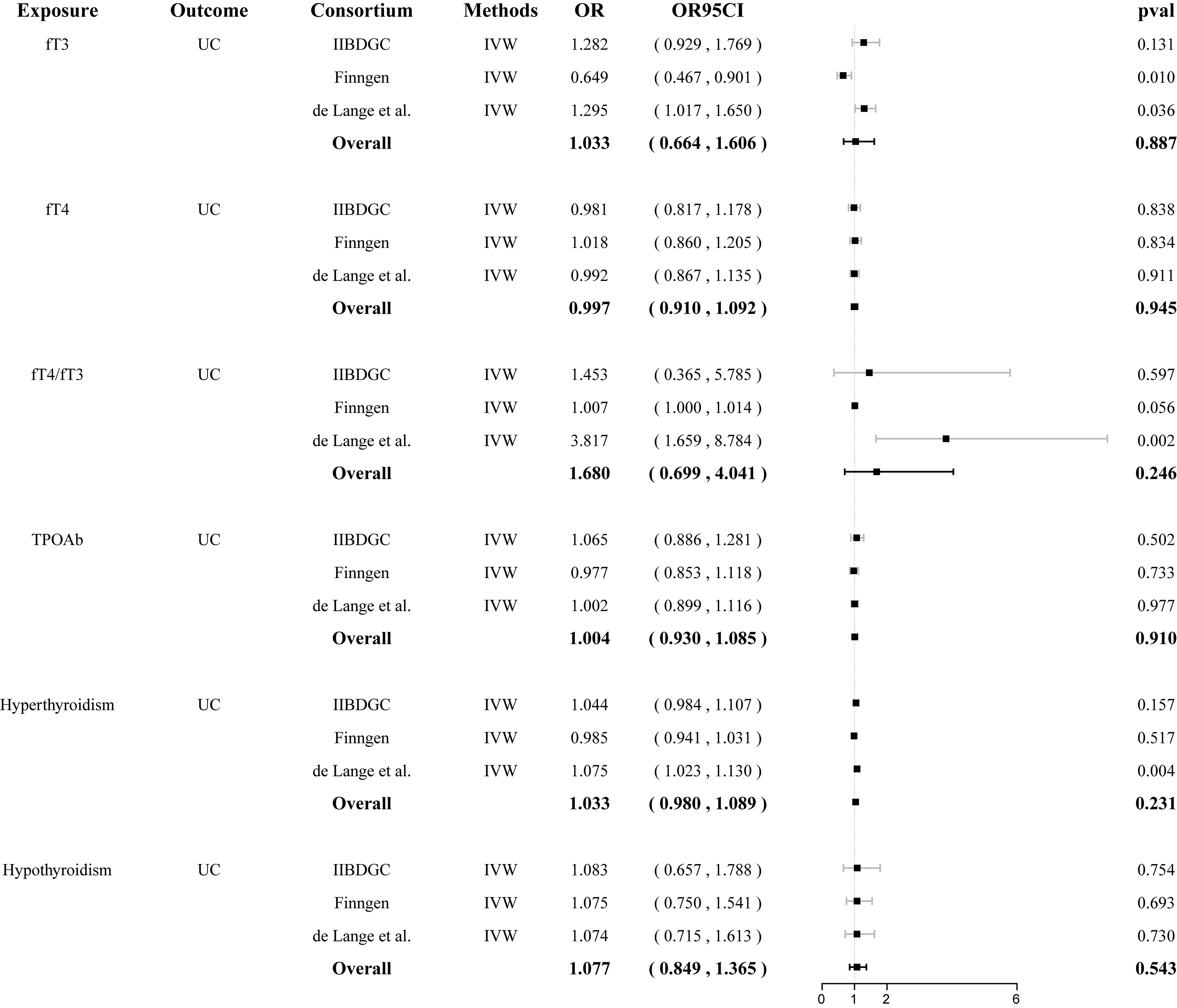

Supplement: Supplementary file 5 [file Image_3.tif]

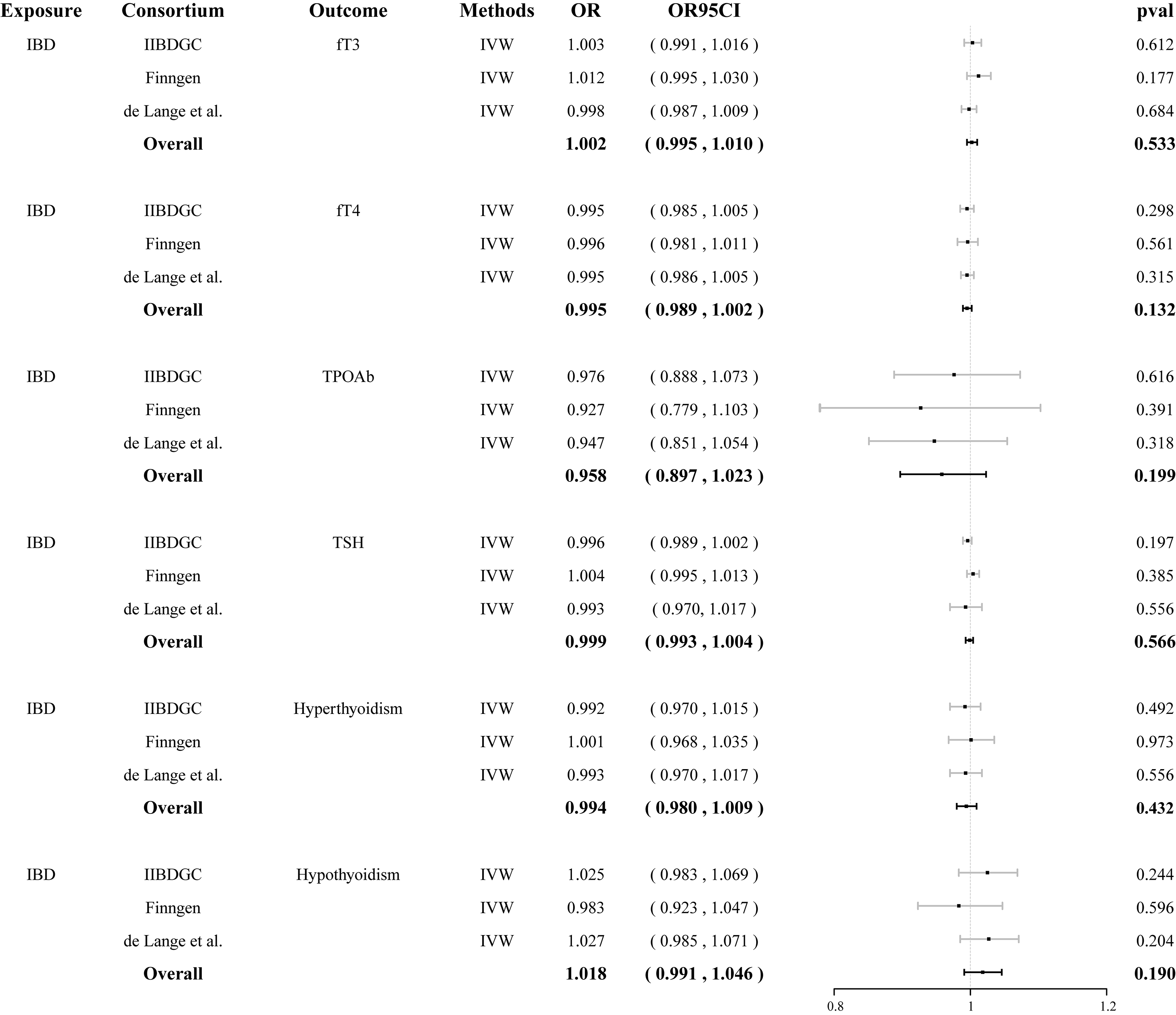

Supplement: Supplementary file 6 [file Image_4.tif]

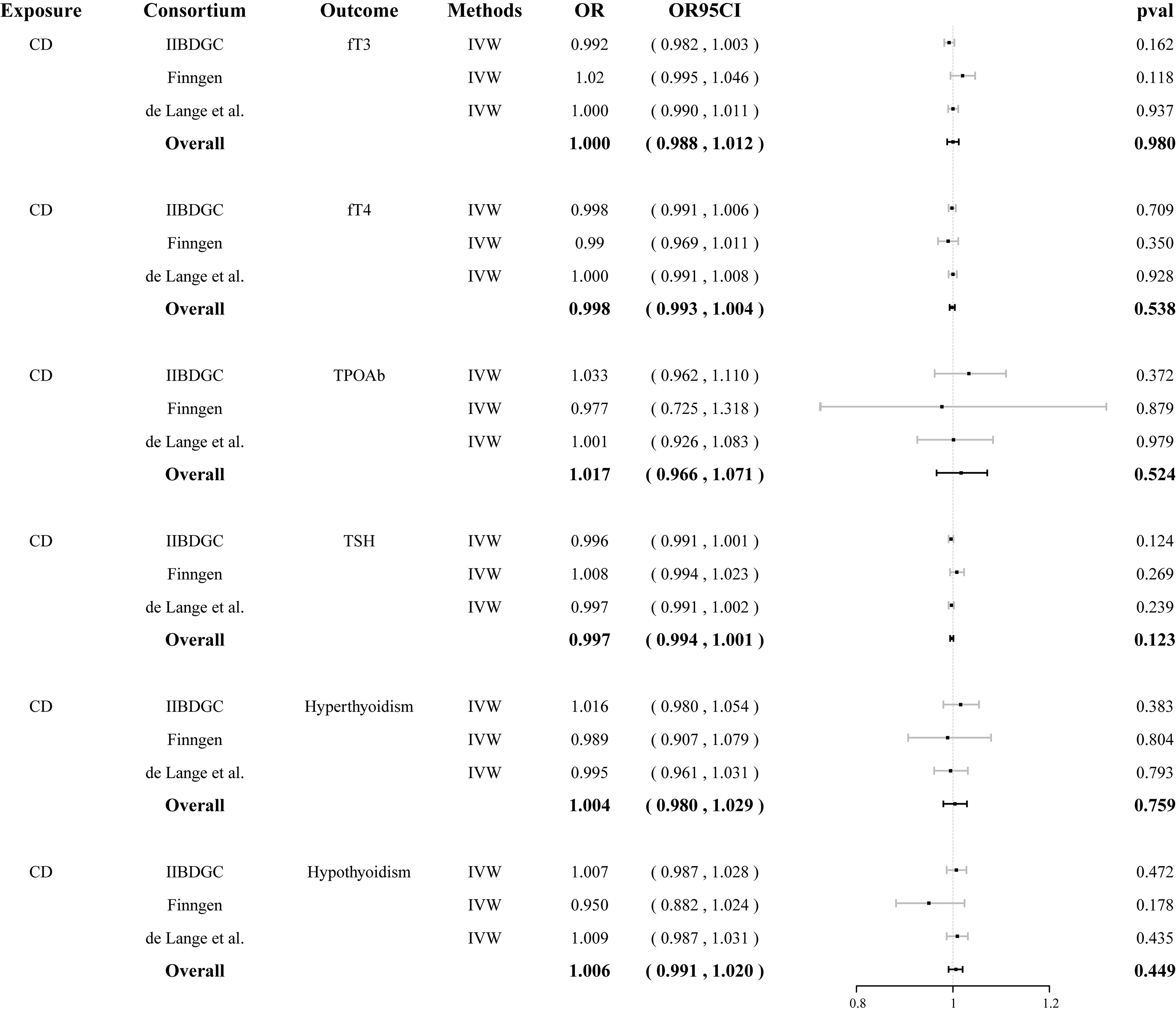

Supplement: Supplementary file 7 [file Image_5.tif]

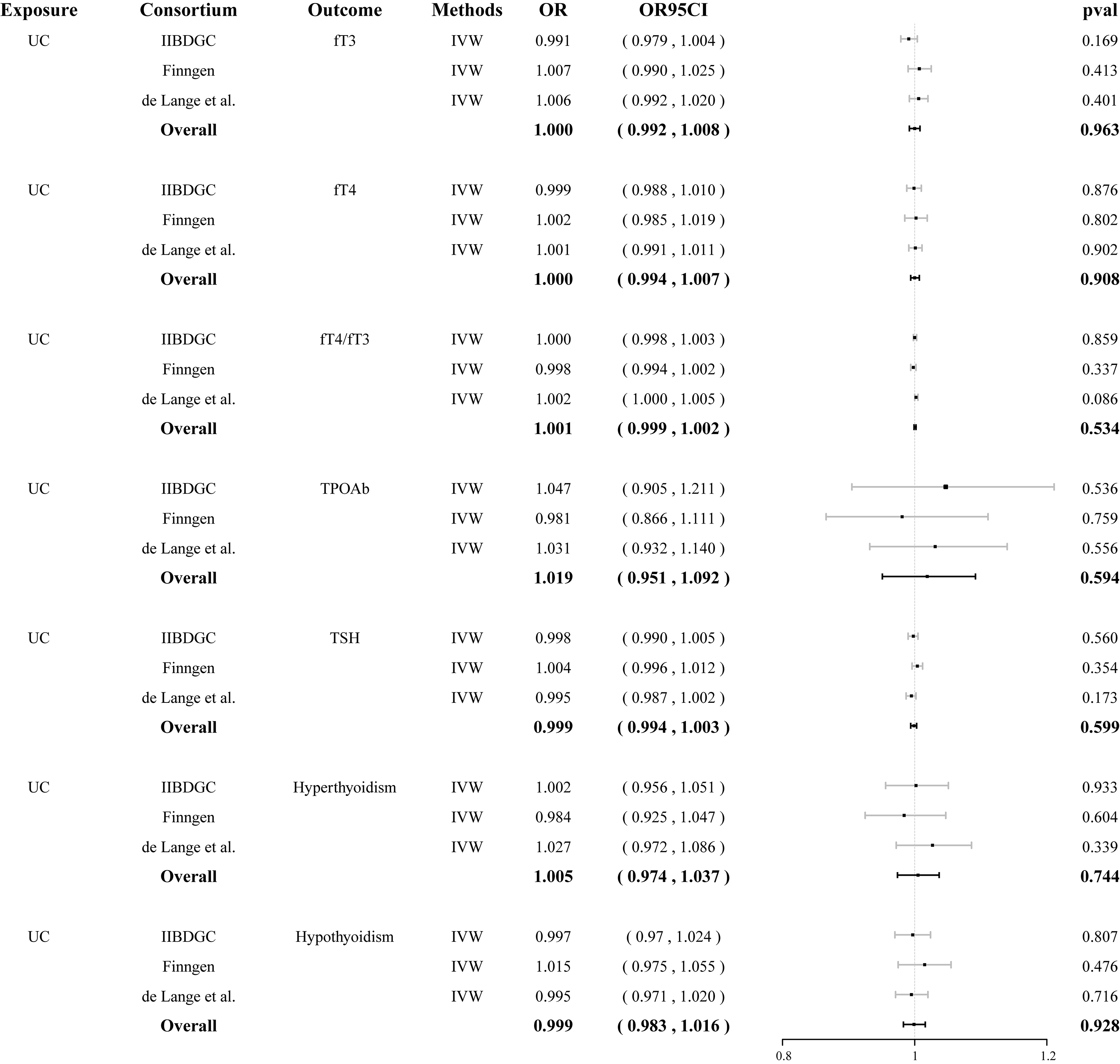

Supplement: Supplementary file 8 [file Image_6.tif]

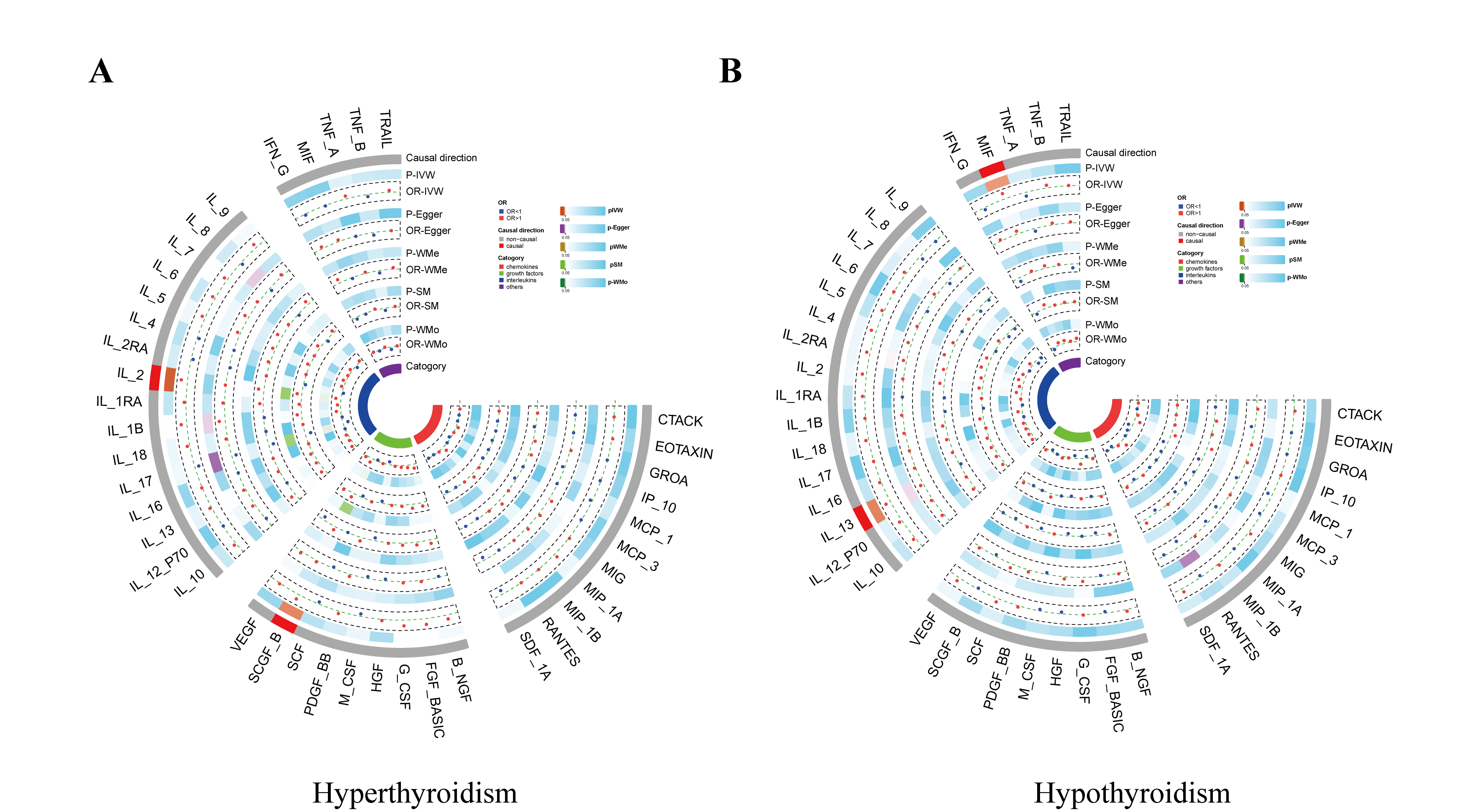

Supplement: Supplementary file 9 [file Image_7.tif]
